# MRI-Based Assessment of Etiology-Specific Sarcopenia Phenotypes in Chronic Liver Disease: A Comparative Study of MASH and Viral Hepatitis

**DOI:** 10.3390/diagnostics16020306

**Published:** 2026-01-17

**Authors:** Mika Yasutomi, Kazuhiro Saito, Yoichi Araki, Katsutoshi Sugimoto, Daisuke Yoshimaru, Shuhei Shibukawa, Masanori Ishida

**Affiliations:** 1 Department of Radiology, Tokyo Medical University, 6-7-1, Nishi-Shinjuku, Shinjuku-ku, Tokyo 160-0023, Japan; mcanaria2424@gmail.com (M.Y.); araki@tokyo-med.ac.jp (Y.A.); d.marumaru@gmail.com (D.Y.); s.shibukawa.rw@juntendo.ac.jp (S.S.); ishida.masanori.2d@tokyo-med.ac.jp (M.I.); 2 Department of Gastroenterology and Hepatology, Tokyo Medical University, 6-7-1, Nishi-Shinjuku, Shinjuku-ku, Tokyo 160-0023, Japan; sugimoto@tokyo-med.ac.jp

**Keywords:** sarcopenia, magnetic resonance elastography, proton-density fat fraction, MASH, viral hepatitis, chronic liver disease

## Abstract

**Background:** Sarcopenia is a clinically important complication of chronic liver disease (CLD), but its underlying mechanisms may differ according to disease etiology. Quantitative MRI biomarkers, including proton density fat fraction (PDFF) and magnetic resonance elastography (MRE), may help characterize etiology-specific patterns of muscle loss. This study aimed to explore etiology-specific associations between MRI-derived biomarkers and sarcopenia, with a particular focus on metabolic dysfunction-associated steatohepatitis (MASH) and viral hepatitis. **Methods:** This retrospective single-center study included 131 CLD patients (77 with MASH, 54 with viral hepatitis) who underwent MRI, including PDFF and MRE. Sarcopenia was defined by L2 skeletal muscle index thresholds (<42 cm^2^/m^2^ for men, <38 cm^2^/m^2^ for women). Muscle identification was performed by automatic threshold-based segmentation by a single observer. Multivariable logistic regression analyses incorporating interaction terms were performed to evaluate whether associations between MRI biomarkers and sarcopenia differed by etiology. **Results:** Sarcopenia was present in 56% of patients. In the overall cohort, older age (OR = 1.05, *p* = 0.01), lower PDFF (OR = 0.93, *p* = 0.03), and lower liver stiffness (OR = 0.51, *p* = 0.006) were independently associated with sarcopenia. A significant interaction between BMI and disease etiology was observed (*p* = 0.02). Subgroup analyses suggested that in MASH, sarcopenia was associated with aging, hepatic fat depletion, and lower stiffness. In contrast, in viral hepatitis, it tended to be associated with higher stiffness and lower BMI. **Conclusions:** MRI-derived hepatic fat and stiffness reflect distinct etiologic patterns of sarcopenia in CLD—metabolically depleted in MASH and fibrosis-related in viral hepatitis. These findings suggest that sarcopenia in MASH and viral hepatitis may reflect different underlying phenotypic patterns, highlighting the importance of considering disease etiology in imaging-based sarcopenia assessment. The results should be interpreted as hypothesis-generating and warrant validation in prospective studies.

## 1. Introduction

Sarcopenia, defined as the progressive loss of skeletal muscle mass and function, has emerged as a critical complication of chronic liver disease (CLD). It is increasingly recognized for its association with poor quality of life, hepatic decompensation, and adverse post-transplant outcomes [1]. While the prevalence of sarcopenia in CLD is well-documented, its underlying pathophysiology is multifactorial and may differ depending on the etiology of liver disease [2].

Metabolic dysfunction-associated steatohepatitis (MASH) and chronic viral hepatitis (HBV/HCV) represent two distinct etiologic categories of CLD, each with unique inflammatory, metabolic, and fibrotic profiles. Sarcopenia in these populations may reflect fundamentally different mechanisms, such as metabolic substrate depletion in MASH versus nutritional deficiency and fibrosis-driven catabolism in viral hepatitis. However, few studies have explicitly addressed etiology-specific risk factors for sarcopenia.

Recent advances in magnetic resonance imaging (MRI) have enabled simultaneous and non-invasive quantification of hepatic steatosis and fibrosis. The proton density fat fraction (PDFF) reflects liver fat content [3,4,5], while magnetic resonance elastography (MRE) provides a reliable estimate of hepatic stiffness, a surrogate marker of fibrosis [6,7,8]. These modalities offer an opportunity to explore mechanistic links between liver pathology and muscle wasting in a more nuanced, quantitative manner.

The purpose of this study was to conduct a comparative, exploratory analysis of etiology-specific associations between MRI-derived biomarkers and sarcopenia in patients with CLD, focusing on MASH and viral hepatitis. We hypothesized that the imaging correlates of sarcopenia would differ between these two etiologies.

## 2. Materials and Methods

### 2.1. Study Design and Population

This retrospective, single-center study was approved by the institutional review board (IRB T2021-0305, 20 March 2022) with a waiver of informed consent. We reviewed electronic medical records and radiology information systems to identify adult patients (≥18 years) with chronic liver disease who underwent liver MRI, including both proton density fat fraction (PDFF) and magnetic resonance elastography (MRE), between January 2021 and December 2024 at our hospital.

**Figure 1:**
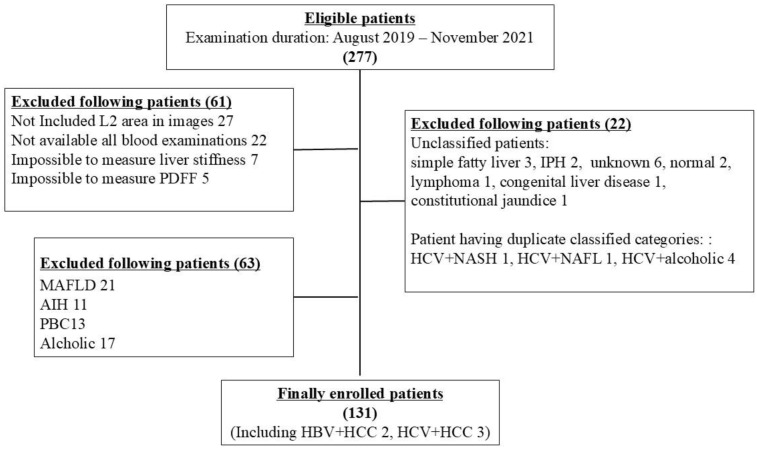
Participant selection flowchart.

Patients were excluded if they had excessive alcohol intake (>20 g/day for women, >30 g/day for men), decompensated cirrhosis (Child-Pugh B or C), or incomplete imaging or clinical data. Hepatologists classified disease etiology into MASH, chronic viral hepatitis B or C (Virus), autoimmune hepatitis, alcoholic liver disease, primary biliary cholangitis, and others. Only patients with MASH or Virus were included for analysis due to a sufficient sample size. Cases with overlapping etiologies (e.g., HCV + NASH), unclear diagnoses, or congenital disorders were excluded. A detailed selection flowchart is shown in Figure 1.

### 2.2. MRI Acquisition and Analysis

The Siemens 3T Skyra (Siemens, Erlangen, Germany) was used. The MRI scans included T1-, T2-, and diffusion-weighted images, and the 6-point DIXON method was utilized for obtaining the PDFF measurements. MRE was performed using a gradient-echo sequence and passive mechanical drivers placed over the liver. Muscle volume measurements were conducted using the 2-point DIXON method. Supplementary Table S1 presents the imaging parameters used for the MRI in this evaluation.

**Figure 2:**
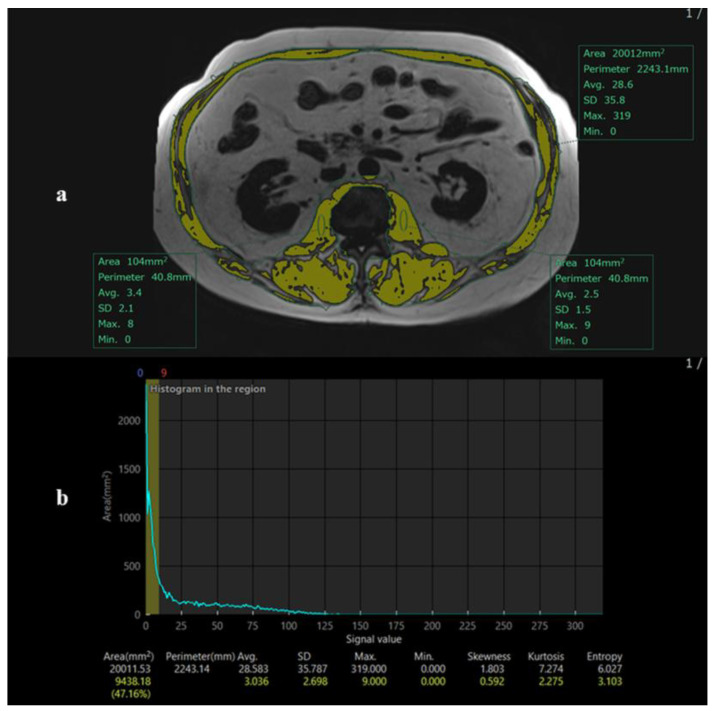
Method for extracting the values of the cross-sectional area for skeletal mass index (L2SMI). (**a**) The method for extracting the values of the cross-sectional area for skeletal mass index (L2SMI) using the 2-point DIXON technique. A circular region of interest (ROI) with an area of 120 mm^2^ is defined within the muscle area that has the highest density, typically the psoas muscle. This ROI is placed in the muscle region of a fat image. The signal intensity within this circular ROI is analyzed. The maximum signal intensity value within the ROI is used as a threshold. Regions within the fat image with signal intensities below the defined threshold are automatically extracted. (**b**) The histogram of the same patient (**a**).

Due to variability in scan ranges, the L2 level was chosen instead of L3, which was not consistently captured. Previous studies, including Wang et al. [9], have demonstrated strong correlations between skeletal muscle area measured at L2 and the conventional L3 level, supporting the validity of this approach. The maximal psoas muscle region was identified, and skeletal muscle area was segmented automatically using intensity-based thresholding (Figure 2). A single observer performed measurements.

Skeletal muscle index (L2SMI) was calculated as muscle area divided by height squared (cm^2^/m^2^) [9]. Sarcopenia was defined as L2SMI < 42 cm^2^/m^2^ for men and <38 cm^2^/m^2^ for women, based on the Japanese Liver Society guidelines [10].

For PDFF, single circular regions of interest (ROI) were placed in the liver parenchyma on the PDFF map, avoiding large vessels, bile ducts, and imaging artifacts. Measurements were usually obtained from the right lobe. For MR elastography, ROIs were placed on stiffness maps within areas of adequate wave propagation and high confidence, avoiding large vessels and liver edges. The mean liver stiffness value was calculated from the selected ROIs.

### 2.3. Clinical Data Collection

Demographic, laboratory, and anthropometric data were extracted from electronic records. Variables included age, sex, platelet count, albumin, AST, ALT, total bilirubin, creatinine, body height, and weight. The Fibrosis-4 (FIB-4) index and albumin-bilirubin (ALBI) score were calculated as noninvasive markers of liver fibrosis and functional reserve, respectively [11,12]. Body mass index (BMI) was also calculated, with a cutoff of 23.0 kg/m^2^ used to define overweight in Asian populations [13].

### 2.4. Statistical Analysis

Continuous variables are expressed as means ± standard deviations or medians (interquartile range), as appropriate. Categorical variables are summarized as frequencies and percentages. Between-group comparisons were performed using Welch’s *t*-test or chi-squared test.

Multivariable logistic regression was performed to estimate odds ratios (ORs) for the presence of sarcopenia.

- Model 1 included the main effects of age, BMI, PDFF, liver stiffness, AST, ALT, and disease etiology.
- Model 2 extended Model 1 by including interaction terms between disease etiology and each continuous predictor (age, BMI, PDFF, stiffness).
- Model 3 and Model 4 were stratified logistic regression models applied separately to MASH and Virus subgroups, respectively, to explore etiology-specific associations. Interaction terms were assessed for statistical significance to determine effect modification by etiology. Model fit was evaluated using the Hosmer–Lemeshow test and pseudo R^2^. Two-sided *p*-values < 0.05 were considered statistically significant. Analyses were conducted using Stata version 18 (StataCorp, College Station, TX, USA).

## 3. Results

### 3.1. Baseline Characteristics

Among 131 patients included in the analysis, 77 had MASH and 54 had chronic viral hepatitis (HBV or HCV). The overall prevalence of sarcopenia was 55.7% (73/131). As shown in Table 1, patients with sarcopenia were significantly older and had lower BMI, PDFF, and liver stiffness than those without sarcopenia.

Stratified by etiology, sarcopenia was observed in 44.9% (35/77) of MASH patients and 70.4% (38/54) of Virus patients (Supplementary Table S2). Patients with viral hepatitis and sarcopenia had notably lower BMI and higher liver stiffness compared to their MASH counterparts.

**Figure 3:**
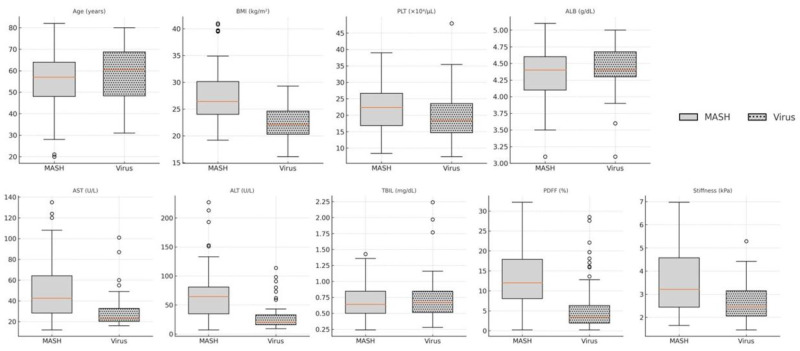
Comparison of baseline variables between MASH and viral hepatitis groups. Boxplots displaying age differences, BMI, platelet count (PLT), albumin (ALB), aspartate aminotransferase (AST), alanine aminotransferase (ALT), hepatic fat content (PDFF), and liver stiffness between patients with MASH and viral hepatitis. Center lines represent medians; box limits indicate the interquartile range (IQR); whiskers denote 1.5 × IQR. Outliers are omitted for clarity. MASH: metabolic dysfunction-associated steatohepatitis. PDFF: proton density fat fraction. Stiffness: MR elastography-derived liver stiffness.

A direct comparison of baseline characteristics between MASH and Virus groups is presented in Figure 3. Patients with viral hepatitis tended to be older, had lower BMI and PDFF, and showed higher liver stiffness, supporting divergent clinical profiles by etiology.

### 3.2. Multivariable Regression by Etiology

Multivariable logistic regression was conducted to identify factors associated with sarcopenia.

Model 1 (overall cohort) showed that older age, lower PDFF, and reduced liver stiffness were independently associated with sarcopenia (Table 2).

Model 2 incorporated interaction terms between disease etiology and each continuous predictor. A significant interaction was found for BMI (*p* = 0.018) and a borderline interaction for liver stiffness (*p* = 0.10), indicating that the effects of these variables on sarcopenia differ by etiology (Table 3, Supplementary Figure S1).

**Figure 4:**
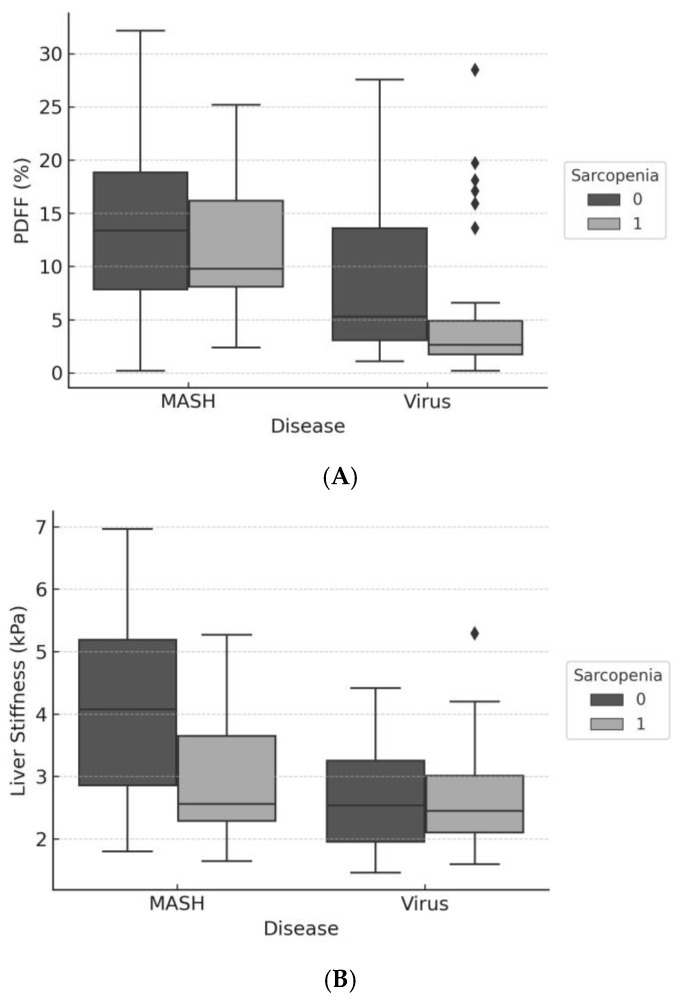
(**A**) PDFF by disease etiology and sarcopenia status. Boxplots comparing hepatic fat content (proton density fat fraction; PDFF) between patients with and without sarcopenia in each disease group (MASH and viral hepatitis). Sarcopenic patients with MASH showed significantly lower PDFF values compared to non-sarcopenic counterparts, suggesting energy substrate depletion. (**B**). Liver stiffness by disease etiology and sarcopenia status. Boxplots showing liver stiffness measurements (kPa) in patients stratified by sarcopenia status and disease etiology. In viral hepatitis, liver stiffness tended to be higher in sarcopenic patients, consistent with advanced fibrosis.

To further explore these differential effects, subgroup analyses were performed. In the MASH subgroup (Model 3), older age, lower PDFF, and lower liver stiffness were significantly associated with sarcopenia (Table 2, Figure 4A,B).

In the Virus subgroup, due to the small number of non-sarcopenic cases (n = 16), a simplified logistic regression model was applied, including only BMI and liver stiffness. In this model, liver stiffness showed a borderline association with sarcopenia (OR = 2.17, *p* = 0.076), while BMI was not statistically significant (*p* = 0.356). These findings are presented in the Supplementary Table S3. These results suggest that sarcopenia in viral hepatitis may be more strongly related to fibrosis burden than to body mass, although confirmation in larger cohorts is warranted.

## 4. Discussion

This study demonstrates that the risk factors for sarcopenia in chronic liver disease differ markedly between patients with MASH and those with chronic viral hepatitis. Using simultaneous MRI-derived measurements of hepatic fat (PDFF) and fibrosis (MRE stiffness), we found that sarcopenia in MASH was associated with aging, hepatic fat depletion, and reduced stiffness, suggesting a metabolic phenotype. Conversely, in viral hepatitis, sarcopenia appeared to be linked to fibrosis burden, although this association was borderline and not statistically significant in simplified regression models.

Importantly, the Virus subgroup had a limited number of non-sarcopenic cases (n = 16), which constrains the statistical power for full multivariable modeling. To mitigate overfitting, we applied a simplified model including only BMI and liver stiffness. In this model, liver stiffness showed a borderline positive association with sarcopenia, while BMI was not significant. These findings suggest a possible role of advanced fibrosis in sarcopenia development among patients with viral hepatitis, consistent with prior literature [14,15]. However, caution is warranted in interpreting these results due to the small sample size.

Taken together, our findings support the concept that sarcopenia in chronic liver disease is not a uniform entity, but rather a syndrome driven by distinct pathophysiological mechanisms depending on the underlying disease etiology. In metabolic liver disease, mitochondrial dysfunction induced by lipotoxic stress has been identified as a key mechanism linking hepatic steatosis to skeletal muscle catabolism [16,17]. The inverse association between PDFF and sarcopenia observed in our MASH cohort may reflect hepatic energy substrate depletion and mitochondrial impairment, leading to bioenergetic failure, increased oxidative stress, and accelerated muscle protein breakdown, as demonstrated in experimental and clinical studies [17,18,19]. In this context, lower liver stiffness in sarcopenic MASH patients may indicate a lesser degree of fibrosis or inflammatory activity, or may reflect the relative underrepresentation of patients with advanced fibrosis in this subgroup.

In contrast, the pattern observed in chronic viral hepatitis is more consistent with muscle loss driven by chronic inflammation, impaired nutritional reserve, and the systemic consequences of advanced fibrosis. Previous studies in cirrhotic populations have shown that elevated liver stiffness measured by MRE (e.g., ≥4 kPa) is significantly associated with sarcopenia and independently predicts hepatic decompensation [15]. Although direct evidence focusing exclusively on HBV- or HCV-infected patients remains limited, prior reviews have underscored the clinical importance of skeletal muscle loss in this population [14]. Our virus-stratified analysis, which demonstrated a borderline positive association between liver stiffness and sarcopenia, aligns with these observations and supports a fibrosis-driven mechanism.

The interaction effects observed in our multivariable model underscore the importance of accounting for disease etiology when assessing sarcopenia risk. Uniform intervention strategies may be insufficient: while resistance training and metabolic modulation may benefit sarcopenic patients with MASH, those with viral hepatitis may require more aggressive nutritional support and antifibrotic therapy. Previous studies have shown that resistance training combined with metabolic interventions effectively improves muscle strength and function in patients with NAFLD/MASH [20,21]. In contrast, in chronic viral hepatitis and cirrhosis, where malnutrition and fibrosis are prevalent, nutritional supplementation (e.g., BCAAs) and antifibrotic strategies have been demonstrated to attenuate sarcopenia and support muscle protein synthesis [14,22]. Findings in the viral hepatitis subgroup should be interpreted as hypothesis-generating due to the limited sample size.

This study has several limitations. Its retrospective design and moderate sample size may limit generalizability. Unmeasured confounders, such as inflammatory markers (e.g., CRP), hormonal status, and physical activity, were not assessed. Inflammatory and nutritional parameters were unavailable in this retrospective design. Subgroup analyses, particularly in viral hepatitis, were limited by sample size and statistical power, and the absence of functional sarcopenia measures limits direct clinical translation. The use of the L2 muscle area instead of L3 may affect comparability with some prior literature [9]; however, our method was adapted to the available scan coverage. Although the L3 level is conventionally used, previous studies have demonstrated that cross-sectional muscle areas at L2 and L3 are highly correlated (r > 0.9) and that L2 can serve as a valid surrogate when full L3 coverage is unavailable [9].

In conclusion, our study highlights distinct etiologic profiles of sarcopenia in chronic liver disease. In patients with MASH, sarcopenia is primarily associated with metabolic depletion, reflected by aging, reduced hepatic steatosis, and lower liver stiffness. In contrast, sarcopenia in chronic viral hepatitis appears to be driven by malnutrition and fibrosis burden. These findings emphasize the need for etiology-tailored strategies in the prevention and management of sarcopenia in CLD. MRI-based phenotyping offers a promising tool to guide personalized interventions.

## Figures and Tables

**Table 1:** Comparison of Variables by Sarcopenia Status.

**Variable**	**No Sarcopenia**	**Sarcopenia**	***p*-Value**
Age	54.05	59.70	0.02 *
PLT	20.91	21.76	0.52
ALB	4.34	4.42	0.24
AST	46.46	38.44	0.08
ALT	59.95	47.98	0.11
TBIL	0.75	0.68	0.23
Creatinine	0.89	0.73	0.22
ALBI_score	−2.98	−3.07	0.15
FIB4_index	1.90	1.90	0.98
BMI	27.69	23.63	0.00 *
PDFF	12.75	8.68	0.005 *
Stiffness	3.65	2.85	0.00 *

* *p* < 0.05.

**Table 2:** Logistic Regression by Etiology Group.

	**Variable**	**β**	***p*** **-Value**	**OR**	**95% CI**
Model 1: Overall	Age	0.04	0.01	1.05	1.01–1.08
	BMI	−0.11	0.07	0.89	0.80–1.01
	PDFF	−0.07	0.03	0.93	0.87–0.99
	Stiffness	−0.68	0.006	0.51	0.31–0.82
	AST	−0.002	0.92	1.0	0.96–1.03
	ALT	0.01	0.26	1.01	0.99–1.03
	Disease	−0.005	0.99	0.99	0.37–2.68
Model 3: MASH	Age	0.07	0.006	1.07	1.02–1.13
	BMI	−0.01	0.87	0.99	0.86–1.14
	PDFF	−0.10	0.03	0.91	0.83–0.99
	Stiffness	−1.19	0.0009	0.3	0.15–0.61
	AST	0.007	0.71	1.01	0.97–1.05
	ALT	0.01	0.23	1.01	0.99–1.04
Model 4: Virus	Age	0.02	0.62	1.02	0.96–1.08
	BMI	−0.48	0.003	0.62	0.45–0.85
	PDFF	0.04	0.57	1.04	0.91–1.18
	Stiffness	0.36	0.53	1.44	0.47–4.40
	AST	−0.07	0.27	0.93	0.83–1.05
	ALT	0.01	0.72	1.01	0.94–1.10

**Table 3:** Interaction Effects with Disease Etiology (Model 2).

**Interaction Term**	**β**	***p*-Value**	**OR**	**95% CI**
Age: Disease	−0.04	0.25	0.96	0.89–1.03
BMI: Disease	−0.39	0.02	0.68	0.49–0.93
PDFF: Disease	0.07	0.31	1.07	0.94–1.22
Stiffness: Disease	0.89	0.10	2.42	0.84–7.01

## Data Availability

Data supporting the findings of this study are available from the corresponding author upon reasonable request.

## Supplementary Materials

The following supporting information can be downloaded at: https://www.mdpi.com/article/10.3390/diagnostics16020306/s1. Figure S1: Interaction Effects with Disease Etiology; Table S1: Imaging Parameters of MR Elastography, Proton Density Fat Fraction, and 2-point DIXON; Table S2: Prevalence of Sarcopenia by Disease Etiology; Table S3: Logistic Regression in Virus Group (Simplified Model).
