# Supplementary material for: MRI-Based Assessment of Etiology-Specific Sarcopenia Phenotypes in Chronic Liver Disease: A Comparative Study of MASH and Viral Hepatitis
Source: Diagnostics (Basel). 2026 Jan 17;16(2):306. doi: 10.3390/diagnostics16020306 (PMC12839808; doi:10.3390/diagnostics16020306)
Supplement: Supplementary file 1 [file diagnostics-16-00306-s001.zip › Supplementary Table S1.pdf]

Supplementary Table S1.

Imaging Parameters of MR Elastography, Proton Density Fat Fraction, and 2-point DIXON.

	TR[ms]	TE[ms]	Slice Thickness [mm]	Flip Angle [°]	Average	FOV[mm]	Matrix Size	Bandwidth [Hz/pixel]	Phase Offset	MEG frequency[Hz]
T1_vibe_dixon_tra	4.2	1.34/2.57	2	10	1	360x240	320x224	1040		
vibe_q dixon	9	1.05/2.46/3.69/4.92/6.15/7.38	3.5	4	1	450x400	160x128	1080		
2D-Elastography	1200	47	5		1	400x400	100x100	2174	4	60
