# Supplementary material for: MRI-Based Assessment of Etiology-Specific Sarcopenia Phenotypes in Chronic Liver Disease: A Comparative Study of MASH and Viral Hepatitis
Source: Diagnostics (Basel). 2026 Jan 17;16(2):306. doi: 10.3390/diagnostics16020306 (PMC12839808; doi:10.3390/diagnostics16020306)
Supplement: Supplementary file 1 [file diagnostics-16-00306-s001.zip › Supplementary Table S2.pdf]

Supplementary Table S2. Prevalence of Sarcopenia by Disease Etiology

Disease	Sarcopenia	No Sarcopenia	Total	Prevalence (%)
MASH	35	42	77	45.5
Virus	38	16	54	70.4
