# Supplementary material for: MRI-Based Assessment of Etiology-Specific Sarcopenia Phenotypes in Chronic Liver Disease: A Comparative Study of MASH and Viral Hepatitis
Source: Diagnostics (Basel). 2026 Jan 17;16(2):306. doi: 10.3390/diagnostics16020306 (PMC12839808; doi:10.3390/diagnostics16020306)
Supplement: Supplementary file 1 [file diagnostics-16-00306-s001.zip › Supplementary Table S3.pdf]

Supplementary Table 3. Logistic Regression in Virus Group (Simplified Model)

Logistic regression analysis of sarcopenia in the Virus group (n = 54), using BMI and liver stiffness as predictors. Due to limited sample size (non-sarcopenic cases = 16), this model was simplified to reduce overfitting. Stiffness showed a borderline association with sarcopenia (p = 0.076), while BMI was not significant.

Variable	Coef.	Std.Err.	z	P>|z|	[0.025	0.975]
Intercept	1.058	2.555	0.414	0.679	-3.949	6.066
BMI	-0.097	0.106	-0.923	0.356	-0.304	0.109
Stiffness	0.775	0.437	1.776	0.076	-0.08	1.631
